# Association Between Electronic Cigarette Use and Risk of Obstructive Sleep Apnea Among Korean Adults: A Cross-Sectional Nationwide Population-Based Study

**DOI:** 10.3390/jcm14113616

**Published:** 2025-05-22

**Authors:** Wonseok Jeong, Min Ji Song, Ji Hye Shin, Ji Hyun Kim

**Affiliations:** 1 Department of Public Health, Graduate School, Seoul National University, Seoul 03080, Republic of Korea; wsjeong22@snu.ac.kr; 2 Department of Neurology, Korea University Guro Hospital, Korea University College of Medicine, Seoul 08308, Republic of Korea; songminji1034@gmail.com (M.J.S.); jhshin@kumc.or.kr (J.H.S.)

**Keywords:** obstructive sleep apnea, smoking behavior, electronic cigarette, STOP-Bang questionnaire

## Abstract

**Background**/**Objectives**: Obstructive sleep apnea (OSA) is known to be closely associated with obesity, cardiovascular diseases, stroke, and mortality, and is a growing public health concern in South Korea. While conventional cigarette smoking is an established risk factor for OSA, the impact of electronic cigarette use on OSA remains poorly understood. This cross-sectional study aimed to investigate the association between electronic cigarette use and the risk of OSA among Korean adults. **Methods**: This study utilized data from the 2019–2023 Korea National Health and Nutrition Examination Survey (KNHANES). Smoking behavior was categorized into four groups: electronic cigarette users (dual users of electronic and conventional cigarettes), conventional cigarette smokers, non-smokers, and ex-smokers. The risk of OSA was assessed using the STOP-Bang questionnaire (score ≥ 5), and multivariable logistic regression was used to examine associations between smoking behaviors and OSA risk, with full adjustment for potential confounders. **Results**: Of the total participants, 3.3% were electronic cigarette users, 15.0% conventional cigarette smokers, 26.6% ex-smokers, and 55.1% non-smokers. Compared to non-smokers, the odds of being at high risk for OSA were significantly elevated among electronic cigarette users (OR = 2.01, 95% CI: 1.21–3.33), conventional cigarette smokers (OR = 1.84, 95% CI: 1.32–2.57), and ex-smokers (OR = 1.70, 95% CI: 1.25–2.30). This association remained significant even when the analysis was restricted to male participants. **Conclusions**: The significant association between electronic cigarette use and increased OSA risk highlights the need for targeted smoking cessation strategies and public health interventions that address the underestimated harms of vaping.

## 1. Introduction

Obstructive sleep apnea (OSA) is a common sleep disorder characterized by repeated episodes of upper airway obstruction during sleep, leading to disrupted breathing and decreased oxygen saturation [1]. If left untreated, OSA can lead to serious health consequences, significantly increasing the risk of cardiovascular disease, stroke, and mortality [2,3,4]. According to a recent review [4], OSA contributes to various cardiovascular risks, including myocardial infarction and heart failure, through mechanisms such as sympathetic activation, oxidative stress, inflammation, and endothelial dysfunction. The prevalence of OSA among middle-aged adults in Asia ranges from 4.1 to 7.5% in men and 2.1 to 3.2% in women [5]. In South Korea, however, the burden appears to be even greater, with a reported prevalence of 15.8% as of 2010. This rate is expected to increase even further due to the strong link between OSA, obesity, and aging [6]. Excess body weight is a major risk factor for OSA, as a higher body mass index (BMI) contributes to upper airway narrowing and obstruction during sleep. Age is another key factor, with research indicating that morbidly obese individuals are 12 to 30 times more likely to develop OSA, and that its severity increases by approximately 25% among those aged 50–59 years [7].

This trend is particularly concerning for South Korea, where both the aging population and obesity rates continue to rise. The proportion of individuals aged 60 and older is projected to grow from 13.7% in 2015 to 28.6% by 2050, while adult obesity rates increased from 29.7% in 2009 to 32.4% in 2015 [8,9]. Given these demographic and health trends, the burden of OSA is likely to rise in South Korea. Therefore, early identification and management of key risk factors are essential to mitigate the potential public health burden posed by OSA.

In the early 1980s, approximately 80% of Korean men smoked; however, this rate declined to 34.0% by 2020 due to increasing awareness of smoking-related health risks [10,11]. Conventional cigarette smoking is now widely recognized as a major risk factor for various diseases, including cerebrovascular, cardiovascular, and other circulatory diseases [12]. In contrast, research on the health effects of electronic cigarettes remains limited, which may be contributing to their growing popularity. Consequently, electronic cigarette use among Korean adults has increased, rising from 2.0% in 2013 to 7.1% in 2015 [13].

Conventional cigarette smoking is a well-established risk factor for OSA, with studies demonstrating a significant association between smoking and increased OSA severity [14,15,16,17]. The underlying mechanisms include upper airway inflammation and collapse driven by calcitonin gene-related peptide-mediated neurogenic inflammation in the uvula, as well as impaired nasal mucociliary function in a dose-dependent manner [17]. Similarly, electronic cigarette use has been associated with adverse health outcomes comparable to those of conventional cigarettes, including diabetes mellitus, dyslipidemia, and hypertension [18,19,20,21,22]. It has also been shown to induce cardiopulmonary changes such as elevated blood pressure, vascular and airway inflammation, and lung damage, suggesting that vaping is not a harmless alternative [23]. Despite the increasing prevalence of electronic cigarette use and the potential overlap in risk factors associated with OSA, research examining the direct relationship between electronic cigarette use and OSA risk remains limited [13,16]. A previous study identified an association between dual use of conventional and electronic cigarettes and OSA but did not account for ex-smokers [24], despite the critical role of smoking duration in assessing OSA risk [25]. To address this research gap, it is important to examine the relationship between OSA and smoking behaviors, including both electronic cigarette use and ex-smoker status as key factors. Specifically, this study aimed to investigate the relationship between smoking behavior and the risk of OSA in Korean adults by comparing high-risk and low-risk groups across electronic cigarette users, conventional cigarette smokers, ex-smokers, and non-smokers. We hypothesized that electronic cigarette users, like conventional smokers and ex-smokers, would have a higher risk of OSA compared to non-smokers.

## 2. Materials and Methods

### 2.1. Data and Study Population

This study utilized data from the 2019–2023 Korean National Health and Nutrition Examination Survey (KNHANES), a rigorously designed national surveillance system developed and maintained by the Korea Disease Control and Prevention Agency (KDCA). KNHANES collects comprehensive information on health status and behaviors, including smoking habits, dietary patterns, and the prevalence of chronic diseases. The KNHANES combines self-administered questionnaires, face-to-face interviews, and standardized physical examinations conducted by trained professionals to ensure the accuracy of the data. Ethics approval for the use of KNHANES data was waived by the Institutional Review Board of the KCDA (IRB No. 2018-01-03-C-A) under the Bioethics and Safety Act of 2015, as the dataset is publicly accessible (https://knhanes.kdca.go.kr/knhanes/main.do (accessed on 1 March 2025)).

The KNHANES employs a complex, stratified, multistage probability-cluster sampling method with a rolling sampling design to ensure the representativeness of the non-institutionalized civilian population in South Korea. For geographic stratification, the survey divides the country into 16 provinces. The sampling process involves two stages: first, primary sampling units (PSUs) are created based on sex, 26 age groups, and 24 classifications of land and housing types. Then, 20 households are randomly selected within each PSU. All household members are required to provide written informed consent, ensuring ethical participation. Due to this rigorous methodology, KNHANES data are considered highly reliable and generalizable to the South Korean population [26].

**Figure 1:**
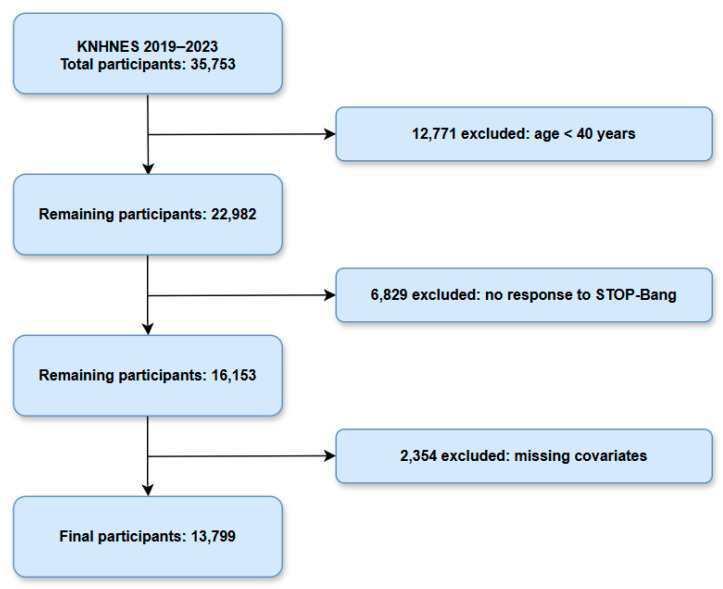
Flowchart depicting the enrollment process of the participants.

Of the 35,753 individuals who participated in the survey, those aged under 40 years (*n* = 12,771) were excluded, as the STOP-Bang questionnaire has not been validated for individuals younger than 40. Among the remaining 22,982 participants, 6829 were excluded due to non-response to the STOP-Bang questionnaire, resulting in a sample of 16,153 participants. Lastly, participants with missing data on key covariates such as drinking status, region, and the presence of diabetes or hypertension, were excluded, yielding a final analytical sample of 13,799 participants (Figure 1).

### 2.2. Independent Variables

Smoking behavior, the key independent variable in this study, was categorized into four groups: electronic cigarette users, conventional cigarette smokers, ex-smokers, and non-smokers. Participants who responded “No” to both the questions “Have you ever smoked a conventional cigarette?” and “Have you ever used an electronic cigarette?” were classified as non-smokers. Those who answered “Yes” to either question were further asked “In the past 30 days, have you smoked or vaped conventional or electronic cigarettes?” Their responses determined their classification into one of the remaining three groups: electronic cigarette users, conventional cigarette smokers, or ex-smokers. The term ‘electronic cigarette’ in this study included both heated tobacco products and liquid-based electronic cigarettes. Dual users of electronic and conventional cigarettes were classified as electronic cigarette users, as only a small proportion of participants (1.44%) reported exclusive use of electronic cigarettes.

The study considered various demographic characteristics, including age groups (40–49, 50–59, and 60 or older), sex, and marital status (married, single, or widowed/separated/divorced). Socioeconomic factors included education level (middle school or below, high school, and college or higher), residential area (urban or rural), household income (low, medium-low, medium-high, and high), and occupation type. Occupational classification was based on the Korean Standard Classification of Occupations, and grouped into four broad categories: white-collar (office work), pink-collar (sales and service jobs), blue-collar (agriculture, forestry, fishery, armed forces), and unemployed [27]. The residential area was categorized as urban or rural based on whether the area was a metropolitan city.

The health-related factors considered in this study included the participants’ drinking status (heavy, moderate, or light alcohol consumption), regular exercise (yes/no), self-reported health status (high, middle, or low), presence of diabetes mellitus (yes/no), hypertension (yes/no), and obesity (yes/no). Light alcohol consumption was defined as abstaining from drinking or drinking once a month or less. Moderate drinkers consumed alcohol approximately two to three times per week, while heavy drinkers drank more frequently. Regular exercise was defined as engaging in weight training at least twice per week. Diabetes mellitus and hypertension were identified based on a physician’s diagnosis. Obesity was determined using a BMI threshold of ≥25 kg/m^2^ in accordance with the guidelines established by the Korean Society for the Study of Obesity [28]. Confounder selection was based on previous research examining the relationship between smoking and OSA, focusing on variables commonly identified as relevant covariates in population-based studies [15,16,17,24,29]. Further details on the definitions of covariates are provided in Supplementary Table S1.

### 2.3. Dependent Variable

The primary dependent variable in this study was the risk of OSA, which was assessed using the STOP-Bang questionnaire, a widely validated screening tool for OSA. Although overnight polysomnography is considered the gold standard for diagnosing OSA, it is resource-intensive, costly, and requires specialized supervision [30]. As a practical alternative, the STOP-Bang questionnaire was developed in 2008 and has since been recognized as a simple, reliable, and effective screening tool for identifying individuals at risk of OSA [31]. The questionnaire comprises eight items, each scored as one point if the condition is met [32]. These conditions include the following: (1) loud snoring, (2) daytime tiredness, (3) witnessed cessation of breathing during sleep, (4) high blood pressure or use of antihypertensive medication, (5) BMI ≥ 35 kg/m^2^, (6) age > 50 years, (7) neck circumference > 40 cm, and (8) male sex. In this study, participants with STOP-Bang scores ≥ 5 were classified as high risk for OSA, while those with scores < 5 were categorized as low risk [24,32].

### 2.4. Statistical Analysis

Chi-square tests were used to assess the general characteristics of the study population. To examine the association between smoking behavior and OSA risk, multivariable logistic regression analysis was performed, adjusting for potential confounders, including demographic, socioeconomic, and health-related factors. Results are reported as odds ratios (ORs) with corresponding 95% confidence intervals (CIs). Statistical significance was defined as a *p*-value < 0.05. All analyses were conducted using SAS software (version 9.4; SAS Institute Inc., Cary, NC, USA).

## 3. Results

**Figure 2:**
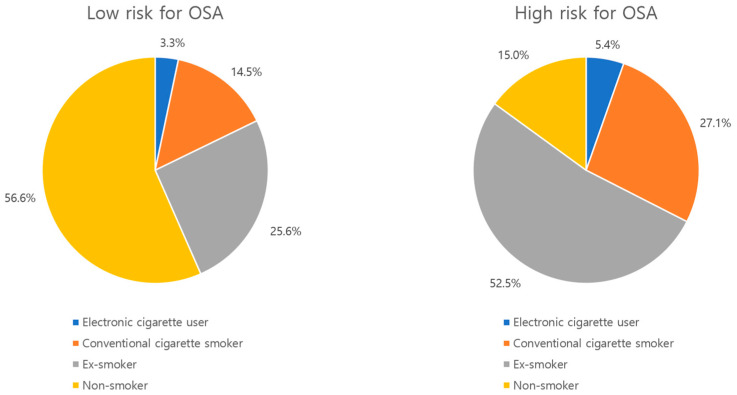
Pie chart showing the distribution of smoking behaviors by OSA risk category.

Table 1 presents the general characteristics of the study population. Based on STOP-Bang scores, 520 out of 13,799 participants (3.8%) were classified as high risk for OSA, while 13,279 (96.2%) were classified as low risk. Among all participants, 3.3% were electronic cigarette users, 15.0% were conventional cigarette smokers, 26.6% were ex-smokers, and 55.1% were non-smokers. Of the 520 participants classified as high risk for OSA, 486 (93.5%) were male (Figure 2).

Table 2 presents the association between smoking behavior and the risk of OSA among study participants after adjusting for relevant covariates. Compared to non-smokers, conventional cigarette smokers and ex-smokers had significantly higher odds of being at high risk for OSA (conventional cigarette smoker: OR = 1.84, 95% CI = 1.32–2.57; ex-smokers: OR = 1.70, 95% CI = 1.25–2.30). Notably, electronic cigarette users exhibited the highest odds of OSA risk (OR = 2.01, 95% CI = 1.21–3.33). As expected, male participants were significantly more likely to be at high risk for OSA compared to females (OR = 10.06, 95% CI = 6.58–15.40). Obesity was also strongly associated with elevated OSA risk (OR = 2.86, 95% CI = 2.34–3.48). In addition, participants with hypertension or diabetes mellitus had greater odds of OSA than those without these conditions (hypertension: OR = 2.37, 95% CI = 1.95–2.87; diabetes mellitus: OR = 1.24, 95% CI = 1.01–1.53). These results were all statistically significant after full adjustment for potential confounders.

Table 3 presents robustness checks for the association between smoking behavior and the risk of OSA. To further validate our findings, we re-estimated the multivariable logistic regression models using only male participants, given that men are more susceptible to OSA and more likely to engage in smoking. The results remained statistically significant and the effect sizes were largely consistent with the main analysis (ex-smoker: OR = 1.68, 95% CI = 1.22–2.31; conventional cigarette smoker: OR = 1.77, 95% CI = 1.25–2.51; electronic cigarette user: OR = 1.92, 95% CI = 1.15–3.21). These results were all statistically significant after full adjustment for potential confounders.

## 4. Discussion

### 4.1. Comparison with Prior Literature

Due to the lack of authoritative information regarding electronic cigarette vaping compared to conventional cigarette smoking, smokers often underestimate the potential adverse effects of vaping. In this study, we explored the association between electronic cigarette use and the risk of OSA, using demographic, socioeconomic, and health-related variables gained from the 2019–2023 KNHANES dataset.

Although electronic cigarette use has become increasingly common, only a few studies have explored its potential link to OSA. A prior study reported a positive association between dual use of conventional and electronic cigarettes and elevated OSA risk [24], but it did not account for previous smoking history, an important confounder in evaluating OSA risk [25]. Another study conducted among U.S. adults also found a positive association between electronic cigarette use and increased OSA risk in univariable analysis [29]. However, this association became statistically insignificant in a multivariate analysis, possibly due to adjustment for sex. In contrast, our analysis demonstrated that electronic cigarette use was significantly associated with an increased risk of OSA, even after adjusting for ex-smoker status and other covariates. To enhance the robustness of our findings, we conducted a sensitivity analysis excluding female participants, due to known underreporting bias in self-reported smoking data and substantial sex-based differences in OSA risk. Previous research has shown a discrepancy between self-reported smoking rates and urinary cotinine-based estimates: while 47.8% of Korean men and 6.6% of women self-reported smoking, biomarker analysis revealed higher actual smoking rates of 52.2% in men and 14.5% in women [33]. Furthermore, a systematic review of studies from various countries—including those in Europe, North America, Australia, Latin America, East Asia, and South Asia—reported that the prevalence of OSA ranges from 13% to 33% in men and from 6% to 19% in women [34]. Even when the analysis was restricted to male participants, electronic cigarette use remained significantly associated with increased OSA risk.

Conventional cigarette smoking has long been linked to a range of harmful health outcomes, prompting the South Korean government to implement strict anti-smoking policies [35]. Consequently, many smokers have turned to electronic cigarettes as a perceived safer alternative. However, despite being marketed as an effective smoking cessation aid, electronic cigarettes remain controversial, with limited experimental evidence supporting their efficacy [36]. In 2015, Public Health England released a statement endorsing electronic cigarettes as a harm reduction strategy [37]. However, this position was based largely on a single study that has since been criticized for bias and methodological limitations, raising concerns about the reliability of the report [38]. Our findings contribute to the growing body of evidence suggesting that electronic cigarette use is not a harmless alternative. Electronic cigarette users in our study had a higher risk of OSA compared to both non-smokers and ex-smokers, emphasizing the need for evidence-based cessation strategies that prioritize complete abstinence from smoking.

The results from self-reported health status support the notion that electronic cigarette vaping is associated with an increased risk of OSA. Participants who used electronic cigarettes were more likely to rate their health as “low” aligning with previous studies indicating that health concerns are a key motivator for smoking cessation [39]. Those reporting lower health status were also more likely to be in the high-risk group for OSA, further reinforcing the association between vaping and poor health outcomes. The lack of an observed association between alcohol consumption and OSA may be due to limitations in measurement, as self-reported data may fail to capture night-time or pre-sleep drinking patterns that are more directly relevant to sleep-disordered breathing.

### 4.2. Biological Mechanisms

Emerging evidence suggests that electronic cigarette use may be associated with an increased risk of OSA through several medical and chemical mechanisms. Nicotine, a primary component of most electronic cigarettes, has been shown to disrupt sleep architecture by reducing total sleep time and increasing sleep fragmentation, potentially worsening OSA symptoms [40]. In line with these findings, recent studies have reported reduced sleep quality among electronic cigarette users [41,42,43], consistent with earlier evidence observed in conventional cigarette smokers [17,44,45]. Moreover, even nicotine-free electronic cigarettes contain aerosols with substances like propylene glycol and vegetable glycerin, which can cause airway inflammation and increase mucus production, potentially leading to airway obstruction during sleep [46]. The heating of these substances in electronic cigarettes can also generate aldehydes such as acrolein, which induce oxidative stress and respiratory tract inflammation, further contributing to airway dysfunction [47]. Collectively, these factors may increase the risk of developing or exacerbating OSA not only for conventional cigarette smokers but also for electronic cigarette users.

### 4.3. Strengths and Limitations

This study has at least four limitations that should be considered before drawing conclusions. First, because the data are based on self-reported measures, there is potential for recall bias of under-reporting or over-reporting. Second, the cross-sectional design of the study prevents us from establishing causal relationships or determining the direction of the observed associations. Third, the classification of electronic cigarette users included individuals who also used conventional cigarettes, which may confound the observed associations. Lastly, our analysis assessed the association between smoking behavior and the risk of OSA using the STOP-Bang score, a screening tool rather than a definitive diagnostic test for OSA.

Despite these limitations, our study has notable strengths. The KNHANES is a nationally representative survey conducted using stratified cluster sampling, which ensures the statistical reliability of the data and the high generalizability of the results. This study utilized five years of KNHANES data, providing a larger and more robust sample than previous studies that typically examined only one or two years. The dataset includes comprehensive health interviews, physical examinations, and nutrition assessments, offering a strong foundation for the development of national health policies and programs. To our knowledge, this is the first study to examine the relationship between electronic cigarette use and OSA risk while adjusting for ex-smoker status using the STOP-Bang questionnaire, an established tool for assessing OSA risk in the general population. Our findings underscore the potential health risks of electronic cigarette vaping and suggest that public health strategies should encourage complete cessation and support evidence-based quitting methods.

### 4.4. Implications

Numerous studies have established that conventional cigarette smoking is strongly associated with various cardiovascular diseases including OSA [12,15,18]. With the introduction of electronic cigarettes, many smokers adopted them under the assumption that they are a safer alternative to traditional cigarettes [48]. While electronic cigarettes are often marketed as a harm reduction tool for conventional smokers, emerging evidence presents a more complex picture, with several studies highlighting their potential health risks. Certainly, the long-term health effects of electronic cigarettes remain largely unverified. Given the limited research on the health risks associated with vaping electronic cigarettes, our study aimed to examine the association between electronic cigarette use and the prevalence of OSA. Our findings indicate that individuals who vape are at a higher risk of developing OSA, similar to conventional cigarette smokers when compared to non-smokers and ex-smokers. Our finding that electronic cigarette use is associated with approximately double the odds of high-risk OSA may be clinically meaningful. Similar thresholds have been used in other clinical contexts, such as gestational diabetes screening, to identify individuals who may benefit from further evaluation and intervention [49].

## 5. Conclusions

Using data from a nationwide population-based survey, we have observed that electronic cigarette use was significantly associated with an increased risk of OSA, even after adjusting for key covariates and past smoking status. Considering the anticipated rise in OSA prevalence in South Korea due to population aging and increasing obesity rates, a comprehensive understanding of its risk factors is crucial for effective prevention strategies. Our findings underscore the need for both conventional and electronic cigarette users to consider smoking cessation and call for caution among smokers who view electronic cigarettes as a safer alternative or cessation aid. Future research employing objective measures such as polysomnography and longitudinal study design is warranted to further validate and clarify the causal relationship between electronic cigarette use and OSA.

## Figures and Tables

**Table 1:** General characteristics of the study population.

		Total (*n* = 13,799)		STOP-Bang Score for OSA				
				High Risk (*n* = 520)		Low Risk (*n* = 13,279)		*p*-Value
		*n*	%	*n*	%	*n*	%	
Smoking Behavior	Electronic cigarette user	460	(3.3)	28	(5.4)	432	(3.3)	<0.0001
	Conventional cigarette smoker	2066	(15.0)	141	(27.1)	1925	(14.5)	
	Ex-smoker	3674	(26.6)	273	(52.5)	3401	(25.6)	
	Non-smoker	7599	(55.1)	78	(15.0)	7521	(56.6)	
Age (years)	40–49	3724	(27.0)	45	(8.7)	3679	(27.7)	<0.0001
	50–59	3543	(25.7)	182	(35.0)	3361	(25.3)	
	≥60	6532	(47.3)	293	(56.3)	6239	(47.0)	
Gender	Male	6591	(47.8)	486	(93.5)	6105	(46.0)	<0.0001
	Female	7208	(52.2)	34	(6.5)	7174	(54.0)	
Educational level	Middle school or less	3882	(28.1)	130	(25.0)	3752	(28.3)	0.1558
	High school	4529	(32.8)	188	(36.2)	4341	(32.7)	
	College or over	5388	(39.0)	202	(38.8)	5186	(39.1)	
Marital status	Married	10,767	(78.0)	442	(85.0)	10,325	(77.8)	0.0004
	Separated or divorced	758	(5.5)	57	(11.0)	21	(0.2)	
	Unmarried	2274	(16.5)	21	(4.0)	57	(0.4)	
Region	Urban area	5724	(41.5)	188	(36.2)	5536	(41.7)	0.012
	Rural area	8075	(58.5)	332	(63.8)	7743	(58.3)	
Household income level	Low	2592	(18.8)	100	(19.2)	2492	(18.8)	0.5315
	Lower middle	3403	(24.7)	114	(21.9)	3289	(24.8)	
	Upper middle	3754	(27.2)	147	(28.3)	3607	(27.2)	
	High	4050	(29.3)	159	(30.6)	3891	(29.3)	
Occupational classification	White-collar	3103	(22.5)	128	(24.6)	2975	(22.4)	0.0018
	Pink-collar	1745	(12.6)	55	(10.6)	1690	(12.7)	
	Blue-collar	3723	(27.0)	171	(32.9)	3552	(26.7)	
	None	5228	(37.9)	166	(31.9)	5062	(38.1)	
Self-Reported Health Status	High	4145	(30.0)	116	(22.3)	4029	(30.3)	<0.0001
	Middle	6858	(49.7)	229	(44.0)	6629	(49.9)	
	Low	2796	(20.3)	175	(33.7)	2621	(19.7)	
Alcohol Consumption	Heavy	1001	(7.3)	82	(15.8)	919	(6.9)	0.8436
	Moderate	5001	(36.2)	240	(46.2)	4761	(35.9)	
	Light	7797	(56.5)	198	(38.1)	7599	(57.2)	
Regular Exercise	Yes	3573	(25.9)	370	(71.2)	9856	(74.2)	0.1171
	No	10,226	(74.1)	150	(28.8)	3423	(25.8)	
Obesity	Yes	5108	(37.0)	340	(65.4)	4768	(35.9)	<0.0001
	No	8691	(63.0)	180	(34.6)	8511	(64.1)	
Hypertension	Yes	2741	(19.9)	233	(44.8)	2508	(18.9)	<0.0001
	No	11,058	(80.1)	287	(55.2)	10,771	(81.1)	
Diabetes	Yes	2483	(18.0)	173	(33.3)	2310	(17.4)	<0.0001
	No	11,316	(82.0)	347	(66.7)	10,969	(82.6)	

Data are expressed as numbers (percentage).

**Table 2:** Factors associated with OSA.

Variables		High Risk of OSA		*p*-Value
		OR	95% CI	
Smoking Behavior	Electronic cigarette user	2.01 *	(1.21–3.33)	0.0069
	Conventional cigarette smoker	1.84 *	(1.32–2.57)	0.0003
	Ex-smoker	1.70 *	(1.25–2.30)	0.0006
	Non-smoker	1.00		
Age (years)	40–49	0.24 *	(0.16–0.35)	<0.0001
	50–59	1.11 *	(0.87–1.42)	0.3849
	≥60	1.00		
Gender	Male	10.06 *	(6.58–15.40)	<0.0001
	Female	1.00		
Educational level	Middle school or less	0.73 *	(0.54–0.99)	0.0397
	High school	0.99	(0.78–1.26)	0.9507
	College or over	1.00		
Marital status	Married	1.51	(0.93–2.45)	0.0988
	Separated or divorced	1.20	(0.70–2.08)	0.5102
	Unmarried	1.00		
Region	Urban area	0.91	(0.75–1.10)	0.3146
	Rural area	1.00		
Household income level	Low	0.95	(0.68–1.32)	0.7476
	Lower middle	0.85	(0.64–1.13)	0.2706
	Upper middle	1.12	(0.8–1.45)	0.3682
	High	1.00		
Occupational classification	White-collar	1.23	(0.90–1.67)	0.1963
	Pink-collar	0.94	(0.73–1.20)	0.6175
	Blue-collar	1.40	(0.98–2.00)	0.0656
	None	1.00		
Self-Reported Health Status	High	0.33 *	(0.25–0.43)	<0.0001
	Middle	0.45 *	(0.36–0.57)	<0.0001
	Low	1.00		
Alcohol Consumption	Heavy	1.52	(1.00–1.35)	0.0051
	Moderate	1.19	(1.00–1.35)	0.1157
	Light	1.00		
Regular Exercise	Yes	0.89 *	(0.72–1.10)	0.0153
	No	1.00		
Obesity	Yes	2.86 *	(2.34–3.48)	<0.0001
	No	1.00		
Hypertension	Yes	2.37 *	(1.95–2.87)	<0.0001
	No	1.00		
Diabetes	Yes	1.24 *	(1.01–1.53)	0.0385
	No	1.00		

Statistical significance was indicated by *. Calculated by adjusted odds ratios (ORs) and 95% confidence intervals (CIs).

**Table 3:** Robustness analysis: factors associated with OSA.

Variables		High Risk of OSA		*p*-Value
		OR	95% CI	
Smoking Behavior	Electronic cigarette user	1.92 *	(1.15–3.21)	0.0124
	Conventional cigarette smoker	1.77 *	(1.25–2.51)	0.0014
	Ex-smoker	1.68 *	(1.22–2.31)	0.0014
	Non-smoker	1.00		
Age (years)	40–49	0.24 *	(0.16–0.36)	<0.0001
	50–59	1.12	(0.87–1.44)	0.3956
	≥60	1.00		
Educational level	Middle school or less	0.69 *	(0.50–0.95)	0.0228
	High school	1.02	(0.80–1.31)	0.8697
	College or over	1.00		
Marital status	Married	1.65	(1.00–2.75)	0.0524
	Separated or divorced	1.24	(0.70–2.22)	0.4614
	Unmarried	1.00		
Region	Urban area	0.89	(0.73–1.09)	0.2641
	Rural area	1.00		
Household income level	Low	1.00	(0.71–1.42)	0.9884
	Lower middle	0.90	(0.67–1.21)	0.4937
	Upper middle	1.14	(0.88–1.48)	0.3213
	High	1.00		
Occupational classification	White-collar	1.33	(0.96–1.84)	0.0835
	Pink-collar	0.98	(0.75–1.27)	0.8752
	Blue-collar	1.70	(1.17–2.49)	0.0058
	None	1.00		
Self-Reported Health Status	High	0.34 *	(0.25–0.45)	<0.0001
	Middle	0.48 *	(0.38–0.61)	<0.0001
	Low	1.00		
Alcohol Consumption	Heavy	1.53 *	(1.14–2.05)	0.0051
	Moderate	1.17	(0.94–1.46)	0.1586
	Light	1.00		
Regular Exercise	Yes	0.87	(0.70–1.08)	0.2112
	No	1.00		
Obesity	Yes	2.82 *	(2.30–3.47)	<0.0001
	No	1.00		
Hypertension	Yes	2.29 *	(1.87–2.80)	<0.0001
	No	1.00		
Diabetes	Yes	1.22	(0.98–1.51)	0.072
	No	1.00		

Statistical significance was indicated by *. Calculated by adjusted odds ratios (ORs) and 95% confidence intervals (CIs).

## Data Availability

The dataset is publicly accessible (https://knhanes.kdca.go.kr/knhanes/main.do (accessed on 1 March 2025)).

## Supplementary Materials

The following supporting information can be downloaded at: https://www.mdpi.com/article/10.3390/jcm14113616/s1, Table S1: Variable Definitions and Classification Criteria.
