# Supplementary material for: Association Between Electronic Cigarette Use and Risk of Obstructive Sleep Apnea Among Korean Adults: A Cross-Sectional Nationwide Population-Based Study
Source: J Clin Med. 2025 May 22;14(11):3616. doi: 10.3390/jcm14113616 (PMC12155413; doi:10.3390/jcm14113616)
Supplement: Supplementary file 1 [file jcm-14-03616-s001.zip › jcm-3625948-supplementary.pdf]

Supplementary Table S1. Variable Definitions and Classification Criteria

**Variables**	**Variable Classification Criteria**
Smoking Behavior	Electronic Cigarette Users, Conventional Cigarette Smokers, Ex-Smoker, Non-Smoker
Age (years)	40-49, 50-59, 60 or greater
Gender	Male, Female
Educational level	Middle school or below, High school, College or higher
Marital status	Married, Single, Widowed/Separated/Divorced
Region	Urban area (metropolitan city), Rural area (otherwise)
Household income level	Low (1st quartile), Lower middle (2nd quartile), Upper middle (3rd quartile), High (4th quartile)
Job Classification	White-collar (office work), Pink-collar (sales and service jobs), Blue-collar (agriculture, forestry, fishery, armed forces), Unemployed
Self-Reported Health Status	High, Middle, Low
Alcohol Consumption	Light alcohol consumption (abstaining from drinking or drinking once a month or less), Moderate alcohol consumption (alcohol approximately two to three times per week), Heavy alcohol consumption (otherwise) (otherwise)
Regular Exercise	Yes (engaging in weight training at least twice per week), No (otherwise)
Obesity	Yes (BMI ≥25), No (otherwise)
Hypertension	Yes (diagnosis of hypertension by a doctor), No (otherwise)
Diabetes	Yes (diagnosis of diabetes by a doctor), No (otherwise)
